# Exploring the Role of a Novel Interleukin-17 Homolog from Invertebrate Marine Mussel *Mytilus coruscus* in Innate Immune Response: Is Negative Regulation by *Mc*-Novel_miR_145 the Key?

**DOI:** 10.3390/ijms24065928

**Published:** 2023-03-21

**Authors:** Xinglu Chen, Longmei Qiu, Xirui Si, Xiaolin Zhang, Baoying Guo, Zhi Liao, Xiaojun Yan, Pengzhi Qi

**Affiliations:** National Engineering Research Center of Marine Facilities Aquaculture, Marine Science and Technology College, Zhejiang Ocean University, Zhoushan 316004, China

**Keywords:** *Mytilus coruscus*, interleukin-17, microRNA, apoptosis, innate immunity

## Abstract

Interleukin-17 (IL-17) represents a class of proinflammatory cytokines involved in chronic inflammatory and degenerative disorders. Prior to this study, it was predicted that an IL-17 homolog could be targeted by *Mc*-novel_miR_145 to participate in the immune response of *Mytilus coruscus*. This study employed a variety of molecular and cell biology research methods to explore the association between *Mc*-novel_miR_145 and IL-17 homolog and their immunomodulatory effects. The bioinformatics prediction confirmed the affiliation of the IL-17 homolog with the mussel IL-17 family, followed by quantitative real-time PCR assays (qPCR) to demonstrate that *Mc*IL-17-3 was highly expressed in immune-associated tissues and responded to bacterial challenges. Results from luciferase reporter assays confirmed the potential of *Mc*IL-17-3 to activate downstream NF-κb and its targeting by *Mc*-novel_miR_145 in HEK293 cells. The study also produced *Mc*IL-17-3 antiserum and found that *Mc*-novel_miR_145 negatively regulates *Mc*IL-17-3 via western blotting and qPCR assays. Furthermore, flow cytometry analysis indicated that *Mc*-novel_miR_145 negatively regulated *Mc*IL-17-3 to alleviate LPS-induced apoptosis. Collectively, the current results showed that *Mc*IL-17-3 played an important role in molluscan immune defense against bacterial attack. Furthermore, *Mc*IL-17-3 was negatively regulated by *Mc*-novel_miR_145 to participate in LPS-induced apoptosis. Our findings provide new insights into noncoding RNA regulation in invertebrate models.

## 1. Introduction

The immune system plays a crucial role in the organism’s defense against the invasion of exogenous pathogens. Conventionally, the defense system is divided into innate immunity and acquired immunity. Innate immunity represents the body’s first line of defense against pathogens, which can detect pathogen invasion and partially eliminate them [1]. Innate immunity is mediated by a great variety of cells, including natural killer cells, monocytes, neutrophils, eosinophils, basophils, and circulating dendritic cells, which are collectively known as innate immune cells. These cells release a large number of cytokines that are involved in cellular communication and thereby help coordinate immune responses [2]. In the case of infection and inflammation, cytokines function as modulators: some cytokines make disease worse (proinflammatory), while others promote health (anti-inflammatory) [3]. Cytokines are subject to high levels of evolutionary pressure and thus exhibit sequence diversification [4], whereas the IL-17 cytokine family shows high conservation, which is manifested by a cysteine-knot fold in the functional architecture formed through interactions among four conserved cysteine residues [5]. Recent studies have demonstrated that IL-17 is a class of proinflammatory cytokines involved in chronic inflammatory and degenerative disorders [6]. The IL-17s function by binding specifically to receptors to promote inflammation development, immune rejection, and hematopoiesis. The IL-17 family of cytokines in humans consists of six members (IL-17A to IL-17F), which are produced by activated T lymphocytes and other innate cell populations in response to IL-1β and IL-23 [7,8]. However, a growing body of evidence suggests that the IL-17 family experienced a marked expansion in marine molluscs and echinoderm species. In the genome of the purple sea urchin *Strongylocentrotus purpuratus*, about 30 IL-17 genes were detected [9]. Similarly, 31 octopus IL-17-like genes were found in *Coleoid cephalopods*, wherein 27 genes have a mighty expression in the suckers and skin [10]. Saco, et al. [11] retrieved 379 unique IL-17 sequences from 15 resequenced mussel genomes and the *M. galloprovincialis* reference genome [12] and divided them into 23 isoforms through phylogenetic analysis. Further, they found that IL-17 isoforms from Mytilidae species were conserved among individuals and shared between closely related species. In addition to Mytilidae species, the large IL-17 families were also found in other molluscs species, including *Crassostrea gigas*, *Mizuhopecten yessoensis*, and *Pinctada fucata martensii* [13]. Seawater is teeming with pathogens, and marine molluscs are in constant contact with them, and therefore a powerful arsenal of immune molecules is needed, thereupon the expansion of the IL-17 family endows these animals with more effective immune responses.

There have been several studies indicating the significant role that IL-17s play in the innate immunity of molluscan animals. For example, research has found that upon infection by *Vibrio harveyi*, the mRNA level of IL-17D was greatly up-regulated in *Tegillarca granosa* [14], while in *C. gigas*, *Cg*IL17-5 showed a distinct reaction to *Vibrio splendidus* [15]. Additionally, molluscan IL-17s have been shown to respond to many pathogen-associated molecular patterns (PAMPs), such as lipopolysaccharide (LPS), polyinosinic:polycytidylic acid (polyI:C), and peptidoglycan (PGN). LPS is a major component of the outer wall of Gram-negative bacterial cell walls and represents one of the most commonly used immune stimulants. On the other hand, polyI:C is a double-stranded RNA analog that is frequently used as a virus simulator in scientific research. After the stimulation by LPS, the transcriptional expression of IL-17 family genes was quickly triggered in the Pacific oyster *C. gigas* and pearl oyster *P. fucata* [16,17]. Also in pearl oyster, *Pf*IL-17 was found to be involved in the immune response to polyI:C stimulation [17]. In addition, a dual luciferase assay showed that *Pf*IL-17 was able to activate vertebrate target genes containing NF-kB binding sites and participate in the NF-kB signaling pathway in HEK293 cells [17]. PGN is present in the cell wall of Gram-positive bacteria and is often used as a mimic for Gram-positive bacteria. The recombinant *C. gigas* IL17-5 was proved to have a strong affinity to PGN, which had never been reported in vertebrate interleukins [18]. These studies provided a prelude to revealing the IL-17 immunomodulatory function in molluscs.

As a class of pro-inflammatory cytokines, the excessive expression of IL-17 can induce serious damage to cells. The organisms have developed various mechanisms to control the overreaction of IL-17, of which microRNAs (miRNAs) represent the most potent and well-studied class of non-coding RNAs. MiRNAs are a family of short RNAs about 22 nt in length that can regulate the expression of target genes by translational repression or mRNA degradation [19]. The current research on the regulation of IL-17s by miRNAs mainly focuses on their synergic effects in human autoimmune diseases such as multiple sclerosis, rheumatoid arthritis, psoriasis, and among others [20]. The technique development of high-throughput sequencing and biological calculation makes it possible to scan molluscan miRNAs at the genomic level, and a large number of conserved and novel miRNAs have been identified from mollusc species such as flat oyster *Ostrea edulis*, Pacific oyster *C. gigas*, *Lymnaea stagnalis*, mussel *M. galloprovincialis*, etc. [21,22,23,24,25,26,27,28,29]. These studies provide basic data and great support for the functional interpretation of miRNAs in molluscs. Concerning the immunoregulatory role of certain miRNAs in molluscs, Tian, et al. [30] found that *Pm*-miR-29a could positively regulate IL-17 in *Pinctada martensii*; after the overexpression of *Pm*-miR-29a, the expression of IL-17 in the mantle and gill of the species was up-regulated. In *C. gigas*, cgi-miR-2d augmented oyster hemocyte phagocytosis by negatively regulating *Cg*IκB2 [31] and negatively regulating the expression of a choline transporter-like gene in the early stage of infection, which was involved in sophisticated immunomodulation [32]. During desiccation, cgi-miR-365 was found to be induced by norepinephrine and directly promoted *Cg*HSP90AA1 expression [33]. Recent studies showed that miRNA scaffold659_26519 targets calmodulin to regulate IL-17 expression in the early phase of the immune response of *C. gigas* [34]. In addition, specific functions of certain miRNAs have been described in other invertebrates, such as sea cucumber *Apostichopus japonicus* [35,36,37,38,39] and prawn *Litopenaeus vannamei* [40]. These studies have opened the veil to the underlying mechanisms of miRNAs in invertebrates and also provided technical and methodological references for our current research.

In our previous study, 26 miRNAs and 667 genes of *M. coruscus* were biologically calculated for differential expression after *Vibrio alginolyticus* challenge, of which *Mc*-novel_miR_145 can target an IL-17 homolog and be involved in the immune response to bacterial infection [41]. The objective of the present study is to identify the IL-17 gene and investigate its potential role in innate immunity and its regulation by the miRNA *Mc*-novel_miR_145 in *M. coruscus*. Through this study, we aim to gain a better understanding of the immune response mechanisms of molluscs and shed light on the molecular basis of their immune defense against pathogens. This research can also provide insights into noncoding RNA regulation in invertebrate models.

## 2. Results

### 2.1. Characterization of McIL-17-3

**Figure 1:**
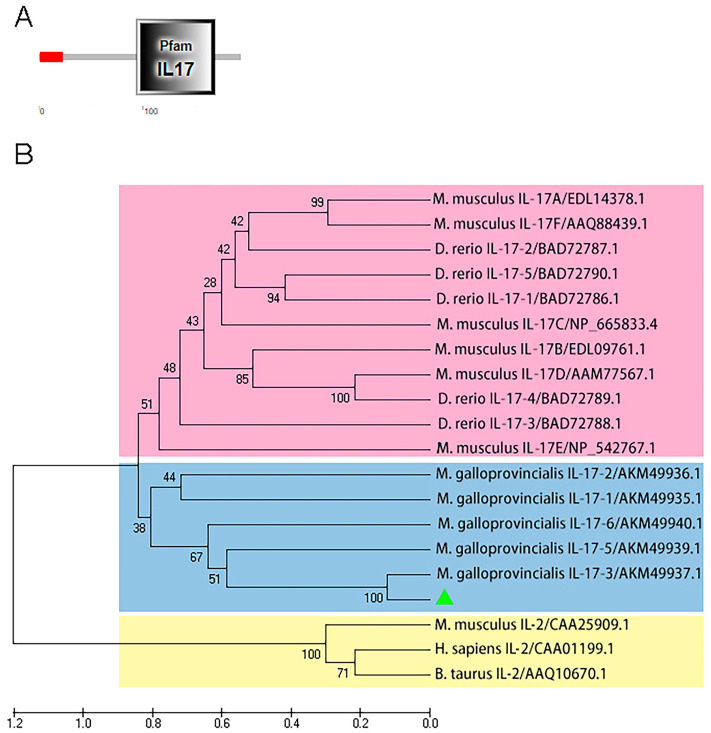
Molecular characterization of *Mc*IL-17-3. (**A**) Architecture analysis of conserved domains in *Mc*IL-17-3 using SMART. A conserved IL-17 domain was shown. (**B**) Phylogenetic analysis of *Mc*IL-17-3. The phylogenetic tree was constructed using MEGAX software with 2000 replications of bootstrapping using the neighbor-joining method. *Mc*IL-17-3 was labeled with a green triangle. Species included in the phylogenetic tree were all retrieved from the Genebank database, and accession numbers were also listed in the tree. Green triangle on behalf of *Mc*IL-17-3. (For interpretation of the references to color in this figure legend, the reader is referred to the Web version of this article.)

The *Mc*IL-17-3 cDNA sequence containing the complete ORF, 3′-UTR, and partial 5′-UTR was in silico cloned from the *M. coruscus* full-length transcriptome (accession number: PRJNA798880, F01_transcript_12404). *Mc*IL-17-3 contains a 585 bp ORF region encoding 194 amino acids. The predicted molecular weight is 21.77 kDa, and the isoelectric point is 5.21. SMART analysis revealed a typical IL-17 domain in this protein (Figure 1A). A phylogenetic tree was constructed by recruiting IL-17 members of vertebrate and Mytilidae species, and IL2s were used as the outgroup. As shown in Figure 1B, these IL-17s gathered into a specific branch to distinguish them from IL-2s. Within the IL-17 cluster, two apparent clades were shown, one composed of vertebrate IL-17s and the other composed of mussel IL-17s. In the mussel IL-17 group, *Mc*IL-17-3 first clustered with the corresponding molecule from another *Mytilus* species, *M. galloprovincialis* (Figure 1B).

### 2.2. Multiple Alignment and Tertiary Structure Prediction

**Figure 2:**
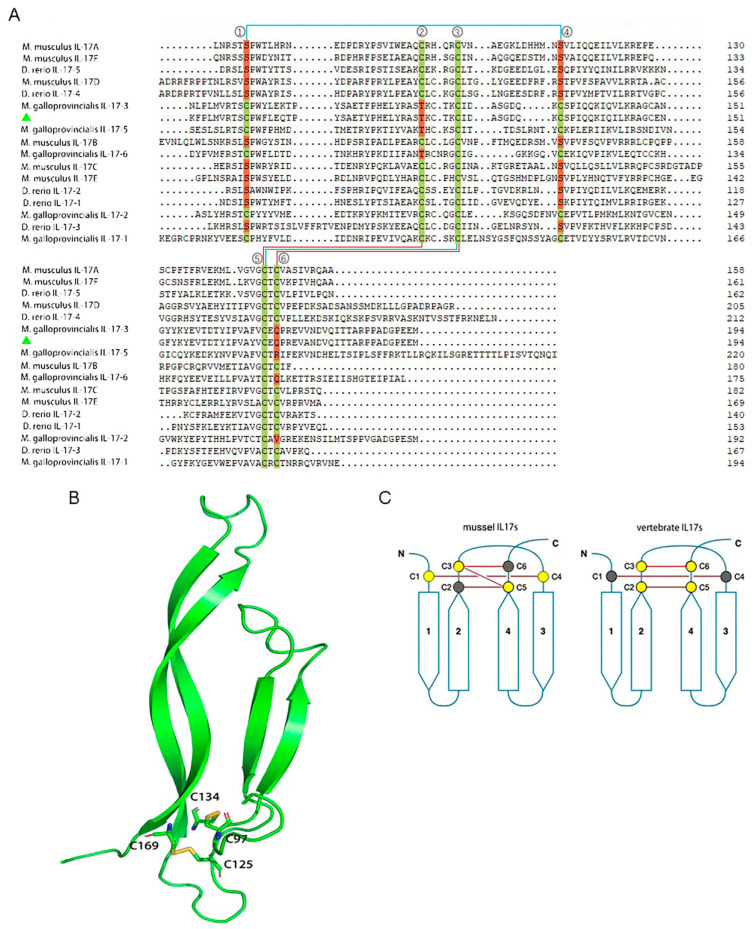
Multiple alignment of *Mc*IL-17-3 with other IL-17 family members. (**A**) The amino acid sequence of *Mc*IL-17-3 was aligned with that of other IL-17s retrieved from mussels and vertebrates, including mice and zebrafish. These cysteines, which form a canonical knot, were marked with green, with replaced amino acid residues marked with red. The cysteines knot is indicated by color lines, and blue meaning their presence in molluscs and red meaning their presence in vertebrates. Note: Only the second half of the sequence alignment is preserved for visualization. (**B**) The tertiary structure of *Mc*IL-17-3 protein was predicted using the Swiss model and pyMol software. These cysteines, which form a canonical knot, were marked. (**C**) A cartoon representation of the canonical cysteine-knot fold. Cysteine residues were indicated by filled circles; those present in IL-17 proteins were yellow, whereas the two missed were gray. (For interpretation of the references to color in this figure legend, the reader is referred to the Web version of this article.)

The core of the *Mc*IL-17-3 is composed of two pairs of antiparallel β-strands; one pair includes strands 1 (residues 52–58) and 2 (residues 66–72 and 77–79), while the other includes strands 3 (residues 89–103) and 4 (residues 110–125) (Figure 2B,C). Two disulfide bridges (Cys97/Cys134 and Cys125/Cys169) connect strands 1 and 3, 2 and 4, respectively (Figure 2A,C). Consistently, other molluscan IL-17s also possess these two disulfide bridges between strands 1 and 3, 2 and 4 (Figure 2A,C). Notably, although vertebrate IL-17s also have two disulfide bridges, they exist between 2 and 4 (Figure 2A,C).

### 2.3. Transcriptional Expression of McIL-17-3

**Figure 3:**
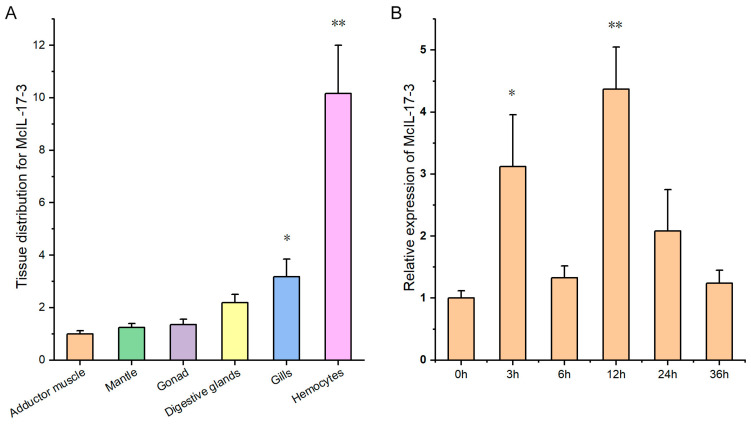
Expression profile analysis of *Mc*IL-17-3 transcripts. (**A**) Distribution of *Mc*IL-17-3 transcripts in common mussel tissues. (**B**) Temporal expression changes of *Mc*IL-17-3 transcripts in response to *V. alginolyticus* challenge. The results were expressed as mean ± SD (*n* = 3, * *p* < 0.05, ** *p* < 0.01). (For interpretation of the references to color in this figure legend, the reader is referred to the Web version of this article.)

The profile of tissue distribution of *Mc*IL-17-3 transcripts was assessed by qPCR. The transcripts of *Mc*IL-17-3 were expressed in all tested tissues, and expression levels in hemocytes and gills were significantly higher than those in adductor muscle (Figure 3A). Temporal expression of hemocyte *Mc*IL-17-3 transcripts in response to *V. alginolyticus* challenge was also assessed. The mRNA level of *Mc*IL-17-3 was significantly up-regulated at 3 and 12 hpc (3.13- or 4.35-fold increase compared to 0 hpc, respectively), but there was no obvious temporal regularity in general (Figure 3B).

### 2.4. The Activation of Downstream by McIL-17-3

**Figure 4:**
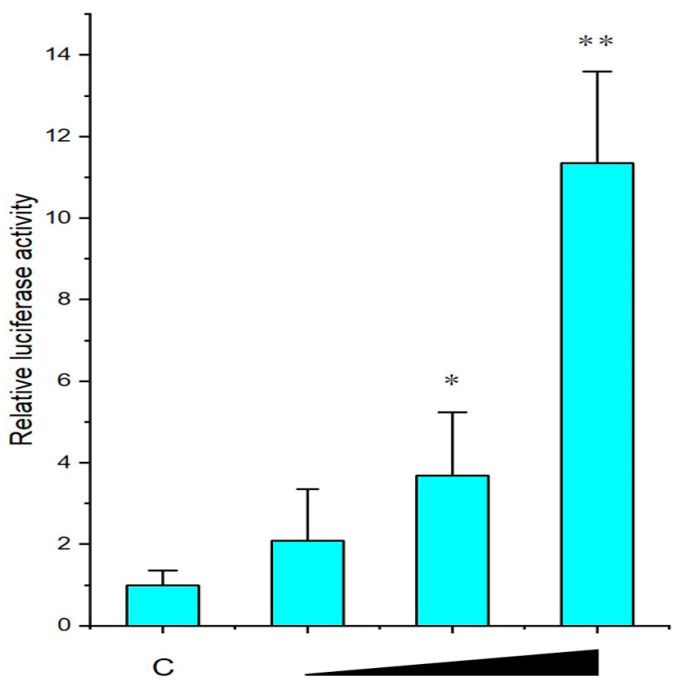
The activation of NF-κB reporter by *Mc*IL-17-3. The recombinant vector *p*EGFP-*Mc*IL-17-3 in three concentrations (0.1, 0.5, and 1.0 µg/well) was cotransfected into HEK293 cells using Lipo6000^TM^ for 24 h. The relative luciferase activities were calculated by normalizing to the pRL-TK value. The experimental results were expressed as fold changes by comparing the luciferase activities of recombinant vector-induced cells with those of empty vector-induced cells at the same concentration. Each value was shown as mean ± SD (*n* = 3), and bars with an asterisk symbol were significantly different (* *p* < 0.05, ** *p* < 0.01).

As shown in Figure 4, recombinant *Mc*IL-17-3 increased the luciferase activity of pGLNF-κB-luc in a dose-dependent manner. At 0.5 and 1.0 µg/well, the luciferase activity of pGLNF-κB-luc increased 2.07- and 11.07-fold, respectively.

### 2.5. Confirmation of McIL-17-3 as a Target Gene of Mc-novel_miR_145

**Figure 5:**
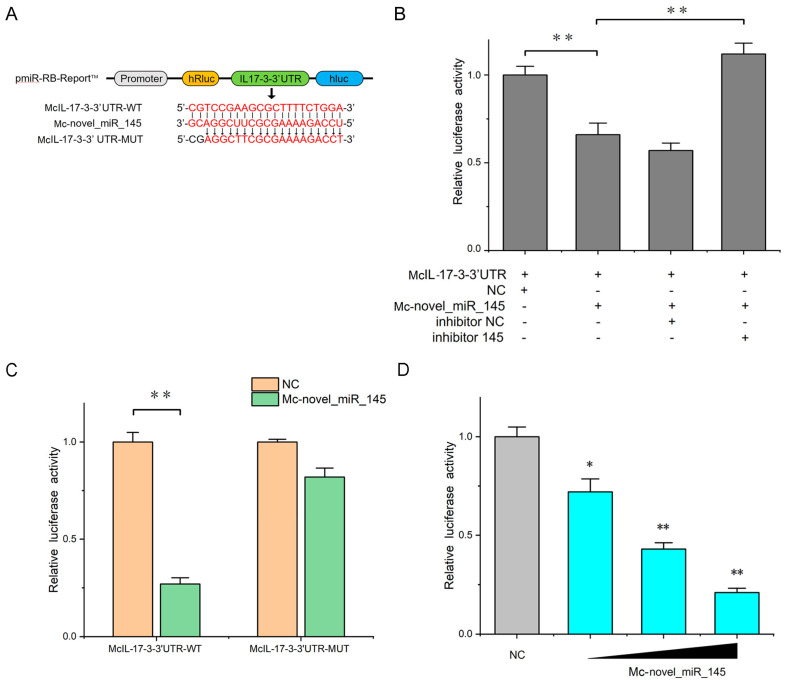
*Mc*-novel_miR_145 targeted *Mc*IL-17-3. (**A**) The *Mc*IL-17-3 3′ UTR sequence was inserted into the pmiR-RB-Report™ vector, respectively constructed wild-type and mutant plasmids. *Mc*-novel_miR_145 sequence and *Mc*IL-17-3-3′UTR target site and mutant site sequence were labeled with red markers. (**B**) *Mc*IL-17-3-3′ UTR-WT plasmid was co-transfected with *Mc*-novel_miR_145 mimic or *Mc*-novel_miR_145 inhibitor into HEK293 cells. (**C**) HEK293 cells were transfected with *Mc*IL-17-3-3′UTR-WT or the mutant type of McIL-17-3-3′UTR-MUT, together with *Mc-*novel_miR_145 or NC, for 24 h. The luciferase activity was measured using the dual-luciferase reporter assay system. (**D**) The concentration gradient experiments were conducted for *Mc*-novel_miR_145 transfection. All data are presented as the means ± SD from at least three independent triplicated experiments. **, *p* < 0.01, *, *p* < 0.05 versus the controls. (For interpretation of the references to color in this figure legend, the reader is referred to the Web version of this article.)

Through bioinformatics prediction, the *Mc*IL-17-3 gene contains a standard target sequence for *Mc*-novel_miR_145 at its 3′UTR (Figure 5A). *Mc*-novel_miR_145 mimic and inhibitor were co-transfected with the wild-type *Mc*IL-17-3-3′UTR reporter plasmid into HEK293 cells to confirm their correlation. As shown in Figure 5B, *Mc*-novel_miR_145 mimic can obviously inhibit the luciferase activity of *Mc*IL-17-3-3′UTR-WT (0.67-fold decrease compared to control), while *Mc*-novel_miR_145 inhibitor remarkably alleviates the effects (1.64-fold increase compared to *Mc*-novel_miR_145 mimic as a merely added group). To assess whether *Mc*-novel_miR_145 directly targets the *Mc*IL-17-3 gene through the target site in the 3′UTR, we constructed the mutant version of luciferase reporter plasmids that mutated the *Mc*-novel_miR_145 targeting sequences in the *Mc*IL-17-3 3′UTR. As shown in Figure 5C, *Mc*-novel_miR_145 mimic significantly decreased the luciferase activity of the cells transfected with the *Mc*IL-17-3-3′UTR-WT (0.27-fold decrease), while no change in luciferase activity was observed in cells transfected with the *Mc*IL-17-3-3′ UTR-MUT. Additionally, the dose-dependent effects of the *Mc*-novel_miR_145 mimic on the inhibition of *Mc*IL-17-3-3′UTR-WT luciferase activity could also be observed at 24 h post-transfection (0.71-, 0.43-, and 0.20-fold decrease compared to control, respectively, Figure 5D).

### 2.6. Mc-novel_miR_145 Negatively Regulates the Expression of McIL-17-3

**Figure 6:**
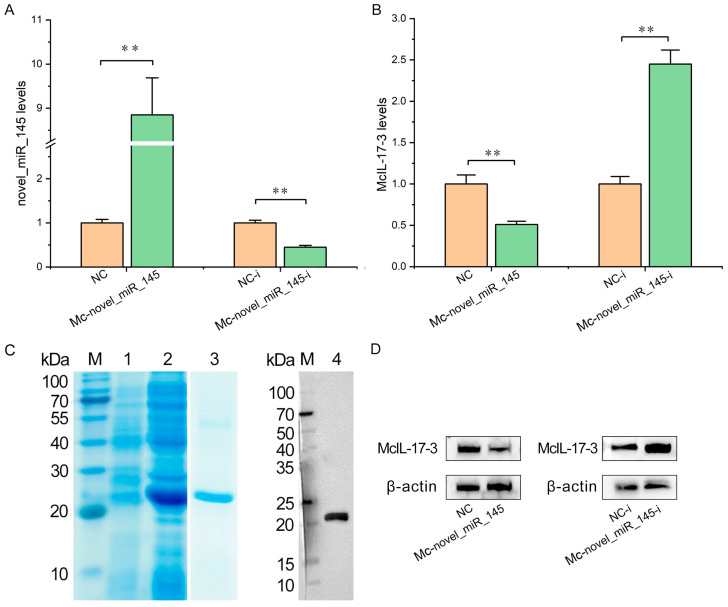
*Mc*-novel_miR_145 inhibited the expression of *Mc*IL-17-3 in *M. coruscus* hemocytes. (**A**) The expression of *Mc*-novel_miR_145 was assessed by qPCR in hemocytes transfected with 145 mimic, 145 inhibitor and their respective control. (**B**) After transfection for 24 h, the transcriptional levels of *Mc*IL-17-3 were determined by qPCR. (**C**) Recombinant expression, purification, and the antiserum preparation for *Mc*IL-17-3. Lane M, standard protein molecular weight marker. Lane 1, negative control (without induction). Lane 2, induced recombinant protein *Mc*IL-17-3. Lane 3, purified *Mc*IL-17-3. Lane 4, Western blot with anti-*Mc*IL-17-3 antibody in the hemocytes of *M. coruscus*. (**D**) After transfection for 24 h, the protein levels of *Mc*IL-17-3 were determined by Western blot. ** *p* < 0.01 versus the controls.

The *M. coruscus* hemocytes were transfected with *Mc*-novel_miR_145 mimic, *Mc*-novel_miR_145 inhibitor, and their respective controls, and the changes in *Mc*IL-17-3 expression were assessed on the transcriptional and protein levels. As shown in Figure 6A, the expression of *Mc*-novel_miR_145 was significantly up-regulated (8.83-fold increase) by its mimic and down-regulated (0.41-fold decrease) by its repressor in hemocytes, suggesting effective effects of these synthetic compound substances. In Figure 6B, the expression of endogenous *Mc*IL-17-3 was significantly inhibited (0.51-fold decrease) by *Mc*-novel_miR_145 mimic at the transcriptional level and significantly induced (2.42-fold increase) by *Mc*-novel_miR_145 inhibitor. To investigate the effects of *M*c-novel_miR_145 on the expression of *Mc*IL-17-3 at the protein level, a polyclonal antibody against *Mc*IL-17-3 was produced. As shown in lanes 2 and 3 in Figure 6C, the recombinant *Mc*IL-17-3 protein was successfully expressed in *E. coli*. The antibody specificity was examined with the mussel protein from hemocytes by Western blot. A single band of about 22 kDa, corresponding to the molecular mass of *Mc*IL-17-3, was observed (lane 4 in Figure 6C). The results of the Western blot in Figure 6D showed the negative regulation of *Mc*IL-17-3 at the protein level *by Mc-*novel_miR_145.

### 2.7. Apoptosis of Hemocytes

**Figure 7:**
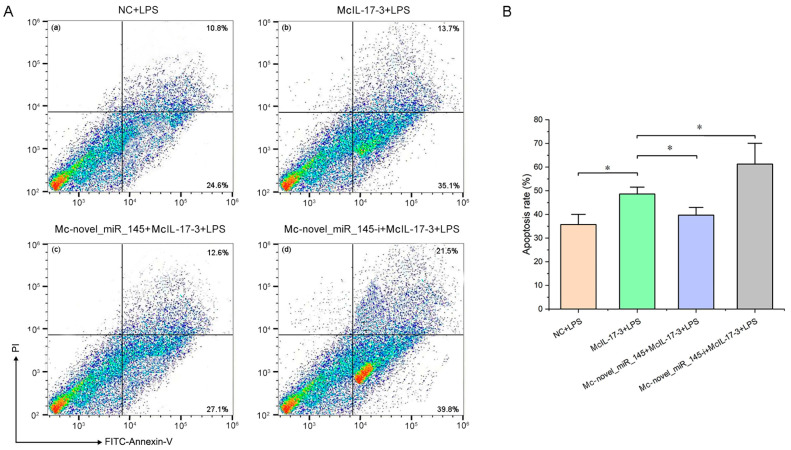
The hemocyte apoptotic rate was assessed by flow cytometry using propidium iodide (PI) and FITC-Annexin-V staining. (**A**) Flow cytometry quadrant diagram. The control group was represented by (**a**): hemocytes with LPS challenge for 24 h. The experimental group by (**b**–**d**) with *p*EGFP-*Mc*IL-17-3 vector, *Mc*-novel_miR_145 + *p*EGFP-*Mc*IL-17-3, and *Mc*-novel_miR_145-I + *p*EGFP-*Mc*IL-17-3 addition. The numbers in the lower right quadrant (FITC^+^/PI^−^) represented the percentage of total fluorescence positive for early apoptosis; and the right upper quadrant (FITC^+^/PI^+^), total fluorescence positive for late apoptosis. (**B**) Significance test of the apoptotic rate. The vertical bars represent the mean ± SD. (*n* = 3, *p* < 0.05 *).

The hemocyte apoptotic rate of four groups, i.e., NC+LPS, *Mc*IL-17-3+LPS, *Mc*-novel_miR_145+*Mc*IL-17-3+LPS, and *Mc*-novel_miR_145-i+*Mc*IL-17-3+LPS was analyzed by flow cytometry using double staining. After *Mc*IL-17-3 was overexpressed, the apoptosis rate of hemocytes challenged with LPS was significantly up-regulated compared to the control group (Figure 7A(a,b),B). When *Mc*IL-17-3 was cotransfected with *Mc*-novel_miR_145, the hemocyte apoptosis rate induced by LPS significantly decreased compared with that of *Mc*IL-17-3 transfected alone (Figure 7A(b,c),B). In contrast, the hemocyte apoptotic rate showed a remarkable increase in the *Mc*-novel_miR_145-i+ *Mc*IL-17-3+LPS group compared to the *Mc*IL-17-3+LPS group (Figure 7A(b,d),B).

## 3. Discussion

IL-17 is recognized to be one of the important pro-inflammatory cytokine families, which can effectively participate in the pathogenesis of different diseases [42]. In our previous study, an *M. coruscus* IL-17 homolog showed differential expression before and after *V. alginolyticus* infection through transcriptome sequencing [41], suggesting its potential role in the innate immune response to bacterial challenge. Here, we characterized this IL-17 homolog and named it *Mc*IL-17-3. The functional domain prediction revealed a typical IL-17 domain, endowing the currently identified gene affiliation to the IL-17 cytokine family. In the phylogenetic tree, this novel IL-17 gene showed a very close kinship with IL-17-3 from another *Mytilus* species, *M. galloprovincialis*. Additionally, they shared a very high 95% amino acid identity, and therefore we nominated the current novel IL-17 gene as *Mc*IL-17-3 to follow the naming convention used in *Mytilus* species. The IL-17 family consists of six members and five receptors in humans [43], but it has experienced a huge genetic expansion in marine invertebrates, especially in mussels [11]. Based on this, our data suggested that *Mc*IL-17-3 was not directly related to certain genes in the human IL-17 family. The cysteine-knot fold located at β-sheets is the typical characteristic of the IL-17 family [5]. Expectedly, the prediction of tertiary structure revealed a cysteine-knot fold in the *Mc*IL-17-3 protein, further deepening the attribution of *Mc*IL-17-3 to the IL-17 cytokine family. To further explore the differentiation of IL-17 amino acid sequences among different species, some typical mussel IL-17s and *Mus musculus* IL-17-A to IL-17-F and *Danio rerio* IL-17-1 to IL-17-3 were selected to perform the multiple alignment analysis. The results showed an interesting finding that the disulfide linkage position of IL-17s in mussels and vertebrates was inconsistent: these two disulfide linkages existed between β-strands 1 and 3, 2 and 4, respectively, in mussels, but only between β-strands 2 and 4 in vertebrates. At the first and fourth cysteine sites, vertebrate IL-17s replace cysteine with serine; contrastingly, at the second cysteine site, mussel IL-17s replace cysteine with threonine; and at the sixth site, with glutamine, arginine, and valine. This is the first time to report the IL-17s differentiation at disulfide linkage in mussels and vertebrates, and its underlying mechanism is unclear and needs further study. Given the polymorphism of IL-17s in mussels, the disulfide linkage between β-strands 1 and 3 may not be as strong as that between 2 and 4, conferring them greater plasticity in mussels. Anyway, analysis of functional domains and tertiary structure suggests that the *Mc*IL-17-3 currently identified is typical of the mussel IL-17 cytokine and might play a similar functional role to its counterparts in molluscs.

The expression of IL-17 genes in human tissues varies widely, with some being expressed in only a few cells and others in a large number of tissues [44,45]. Most of the studies on molluscs showed that IL-17s had a constitutive expression profile [13,15,17,46,47]. Nevertheless, Li, Zhang, Zhang, Xiang, Tong, Qu and Yu [47] showed an inconsistent result in *C. gigas* IL-17s: *Cg*IL-17-2, -3, -4, and -6 were highly expressed in *C. gigas* gills, digestive glands, and mantle, but hardly expressed in other tissues. Here, *Mc*IL-17-3 transcripts were found in all tissues examined, suggesting its multiple functional roles in a variety of physiological activities. However, the high expression levels of *Mc*IL-17-3 in some molluscan immune-related tissues, such as hemocytes, gills, and digestive glands, suggest its closer involvement with immune response. Following, its rapid responsiveness to *V. alginolyticus* attack deepens this point. The injection of *V. alginolyticus* significantly up-regulated the expression of *Mc*IL-17-3, and similar results have also been observed in other molluscan IL-17s. Injection of *V. anguillarum* into *C. gigas* induced a fast increase in *Cg*IL-17 transcript abundance in hemocytes, suggesting that it was an immune early phase gene [46]. When *C. gigas* suffered an attack by another pathogen, *V. splendidus*, the mRNA level of *Cg*IL-17-5 in hemocytes was also significantly elevated [15]. The stimulation of LPS, the major pathogenic component of bacteria, dramatically increased the expression of *Pf*IL-17 in the digestive glands of the pearl oyster *P. fucata* [17]. In addition, LPS could induce the expression of *Cg*IL-17-3 in *C. gigas* [47] and *Pm*IL-17-2 in *P. fucata martensii* [13]. These results collectively indicated that IL-17 played an important role in molluscan immune defense against bacterial attack. In this study, *Mc*IL-17-3 showed the activation capacity of the downstream NF-κB pathway. Similar results were also observed in some mollusc species. In the pearl oyster *P. fucata*, *Pf*IL-17 also exhibited activation of the NF-κB pathway in HEK293 cells by the luciferase reporter assays [17]. In *C. gigas*, *Cg*IL-17-5 promoted the activation of *Cg*MAPKs and the nuclear translocation of *Cg*Rel and *Cg*AP-1 to promote the mRNA expression of cytokines and antibacterial peptides [15]. It has been demonstrated that the mode of action of IL-17 is based on its union into dimers (homodimers or heterodimers), whose activity is highly dependent on their attachment to the receptors (IL-17Rs) they target. These receptors contain a conserved cytoplasmic domain (SEFIR) that interacts with adaptor proteins to initiate downstream signal transduction pathways for activating transcription factors such as NF-κB and promoting the expression of immune and pro-inflammatory target genes, such as cytokines and antimicrobial peptides [11,48,49]. These results suggest that molluscan IL-17s may have the same mode of action as their counterparts in vertebrates. This needs to be confirmed in future studies.

The correlations between miRNAs and IL-17s have been found in several human disease models. Niimoto, et al. [50] confirmed the positive correlation between miR-146a and IL-17a expression in peripheral blood mononuclear cells and synovium from rheumatoid arthritis patients and summarized the vital function of miR-146a in the differentiation of IL-17 producing cells. In psoriasis patients, miR-146a acts as a potent inhibitor of IL-17-driven skin inflammation, and its low levels may contribute to early disease onset in genetically susceptible individuals [51]. MiR-146a may ameliorate periodontitis by down-regulating the expression of IL-17 and inhibiting the proliferation of human periodontal ligament stem cells [52]. MiR-155 negatively regulates the expression of IL-17 to participate in the host immune response to postviral bacterial pneumonia in mice lung; miR-155 inhibited mice show stronger expression of IL-17 in the lung, accompanied by improved bacterial clearance [53]. These studies suggest that IL-17 is regulated by multiple miRNAs and varies with disease and cell type [20]. In a previous study, we performed an integrated analysis of the miRNAome and transcriptome to explore the interactive regulation of miRNA-mRNA by *M. coruscus* in response to *V. alginolyticus* infection. The results predicted that *Mc*-novel_miR_145 could target *Mc*IL-17-3 to participate in the innate immune response to bacterial infection [41]. Aiming to explore the underlying mechanism of *Mc*-novel_miR_145 regulation of *Mc*IL-17-3 in depth, a series of laboratory experiments were conducted in the present studies. The calculation means predicted that *Mc*-novel_miR_145 could target the 3′UTR of *Mc*IL-17-3. The following luciferase reporter assays performed in HEK293 cells co-transfected by *Mc*-novel_miR_145 mimic, mimic NC, inhibitor, inhibitor NC with *Mc*IL-17-3-3′UTR-WT, -MUT reporter plasmids further confirmed this point. Further, *Mc*-novel_miR_145 mimic, mimic NC, inhibitor, and inhibitor NC were transfected into *M. coruscus* hemocytes to evaluate their effects on *Mc*IL-17-3 expression at transcriptional and protein levels. The expression of *Mc*IL-17-3 was significantly down-regulated in the *Mc*-novel_miR_145 mimic transfection group, while significantly up-regulated in the *Mc*-novel_miR_145 inhibitor transfection group, suggesting a negative regulation of *Mc*IL-17-3 by *Mc*-novel_miR_145. The current results were contrary to a previous study. In *Pinctada martensii*, Tian, Zheng, Huang, Jiao and Du [30] found that although *Pm*-miR-29a could target IL-17, the regulation was positive, as the expression of IL-17 in the mantle and gill of *Pinctada martensii* was up-regulated after the overexpression of *Pm*-miR-29a. Given that *P. martensii* and *M. coruscus* both belong to bivalves and are relatively closely related, these inconsistent results suggest that the regulation of IL-17s in molluscs may vary with miRNAs. Other than this, no literature has reported the correlation between miRNAs and IL-17s in molluscs, and therefore there are no more parallel studies to compare the current results. However, a few studies have reported targeting relationships between specific miRNAs and specific immune-related genes in molluscs. For instance, cgi-miR-2d augments oyster hemocyte phagocytosis by negatively regulating *Cg*IκB2 in *Crassostrea gigas* [31]. Additionally, cgi-miR-2d also negatively regulates the expression of one choline transporter-like gene to participate in the sophisticated immunomodulation of oyster hemocytes during the early stage of infection [32]. These studies at least suggest that miRNAs play a potential role in innate immune signaling by targeting specific genes in molluscs, just as they do in vertebrates.

Next, we sought to explore the potential function of the interaction between *Mc*-novel_miR_145 and *Mc*IL-17-3 in the innate immunity of *M. coruscus*, and their role in LPS-induced apoptosis is the focus of our attention. LPS is a highly proinflammatory molecule that is a component of the outer envelope of all Gram-negative bacteria. LPS has been shown to induce apoptosis in various cells and tissues, such as macrophages [54], endothelial cells [55], and mouse lung [56]. A preliminary experiment has demonstrated that LPS exposure at a nominal concentration of 0.1 mg/mL for 24 h significantly induced apoptosis of hemocytes from *M. coruscus*. After *Mc*IL-17-3 overexpression, the apoptosis level of *M. coruscus* hemocytes was significantly increased compared with the control group, suggesting that *Mc*IL-17-3 may strengthen LPS-induced apoptosis of hemocytes in *M. coruscus*. The current results were consistent with some previous studies. In human neutrophils, IL-17A could lessen the anti-apoptotic effects mediated by granulocyte macrophage-colony stimulating factor [57]. IL-17 induces apoptosis of vascular endothelial cells, which is a potential mechanism of acute coronary syndrome [58]. Genetic deletion of IL-17A reduces alveolar type II cell apoptosis and thus alleviates chronic obstructive pulmonary disease [59]. However, there are still some contrary arguments. When mice are infected by Theiler’s murine encephalomyelitis virus, IL-6 and IL-17 synergistically promote viral persistence by inhibiting cellular apoptosis [60]. These conflicts suggest that the underlying mechanism of IL-17-mediated apoptosis is complex, and IL-17s may play opposite functions depending on the type of infection.

When *Mc*IL-17-3 were co-transfected with *Mc*-novel_miR_145, the level of apoptosis induced by LPS was significantly down-regulated compared to the only *Mc*IL-17-3 transfected group, correspondingly, the apoptosis level was significantly up-regulated after *Mc*IL-17-3 was co-transfected with *Mc*-novel_miR_145 inhibitor. *Mc*-novel_miR_145 has been shown to negatively regulate *Mc*IL-17-3, and therefore we concluded that *Mc*IL-17-3 aggravated the hemocyte apoptosis induced by LPS, while *Mc*-novel_miR_145 alleviated the effects. Several miRNAs that have been shown to be involved in human and mouse cell apoptosis were identified in molluscs in the last few years. For instance, miR-125b and miR-335 were found in flat oyster *Ostrea edulis* [21], miR-184 was found in hemocytes of oyster *Crassostrea gigas* [23], and miR-9, -29, -96, -182, and -193 were found in the regenerating central nervous system of the *L. stagnalis* [25]. These apoptosis-related miRNAs identified through high-throughput sequencing and biology calculations confirm the existence of miRNA-mediated apoptosis in molluscs. Chen et al. reported an up-regulation of miR-2d after *Vibrio splendidus* challenge in the hemocytes of *Crassostrea gigas*. The overexpression of miR-2d was correlated with a knocking-down expression of IκB2, and a significant increase in hemocyte phagocytosis rate, linked with a suppression of apoptosis [31]. The results were consistent with our current study, collectively, seeming to imply that miRNAs exert an inhibitory function on cell apoptosis in molluscs. In fact, most of the apoptosis-related miRNAs in humans have shown inhibitory effects on apoptosis. MiR-146 protects A549 and H1975 cells from LPS-induced apoptosis and inflammation injury via up-regulating Sirt1 and thereby blocking NF-κB and Notch pathways [61]. Furthermore, miR-146 attenuates irradiation- and LPS-induced hepatocyte apoptosis through inhibition of the TLR4 pathway [62]. MiR-93 inhibits chondrocyte apoptosis in osteoarthritis by targeting the TLR4/NF-κB signaling pathway [63]. MiR-129-5p alleviates spinal cord injury in mice via suppressing apoptosis through the HMGB1/TLR4/NF-κB pathway [64]. However, there are still some specific miRNAs that show reinforcing actions on apoptosis. For instance, miR-203 was found to accelerate LPS-induced apoptosis by targeting PIK3CA in alveolar epithelial cells [65]. These results suggest the complexity of the underlying mechanism of miRNA-mediated apoptosis.

## 4. Materials and Methods

### 4.1. Experimental Design

Firstly, *Mc*IL-17-3 was identified and characterized from *M. coruscus* through bioinformatic analysis. Following this, the tissue distribution of *Mc*IL-17-3 transcripts as well as its response to bacterial challenge were assessed by quantitative real-time PCR (qPCR) assays. Next, the association between *Mc*IL-17-3 and *Mc*-novel_miR_145 was determined by luciferase reporter analysis performed in HEK293 cells and *M. coruscus* hemocytes. Additionally, their functional role in LPS-induced apoptosis was assessed by flow cytometry.

### 4.2. Artificial Seawater

After three days of aeration of municipal tap water, instant sea crystal (Haiding LTD, Ji’an, China) was added, thoroughly stirred, and melted to reach a salinity of 30‰. The configured artificial seawater needs to be aerated for 2 h before being used.

### 4.3. Animals

The thick-shelled mussel *M. coruscus* (shell length, 9.82 ± 0.53 cm; shell width, 4.68 ± 0.45 cm; wet weight, 71.2 ± 2.7 g) were purchased from Donghe market in Zhoushan, Zhejiang Province, China. All mussels were kept in tanks filled with an ASW of about 25 °C and salinity of 30‰ for more than a week before subsequent experiments.

### 4.4. McIL-17-3 cDNA Identification

An IL-17 homolog (*Mc*IL-17-3) was in silico cloned from the full-length transcriptome of *M. coruscus* (accession number: PRJNA798880, F01_transcript_12404). The Blast procedure was performed to predict the putative *Mc*IL-17-3 amino acid sequence, followed by functional domain analysis with SMART and phylogenetic relationship assessment with MEGA-X. The multiple alignment was performed using the ClustalW procedure. The Swiss model and pyMol were used to predict the tertiary structure of the *Mc*IL-17-3 protein. The detailed procedure was according to our previous study [66].

### 4.5. Quantitative Real-Time PCR Assays

Quantitative real-time PCR assays (qPCR) are a powerful tool for detecting and measuring gene expression levels. Here, the tissue distribution of *Mc*IL-17-3 was evaluated using the qPCR method. In addition, changes in the expression of *Mc*IL-17-3 mRNA in response to bacterial infection were also detected by qPCR. The tissue distribution profile of *Mc*IL-17-3 was determined in gills, mantle, digestive glands, gonad, adductor muscle, and hemocytes using qPCR programmed at 95 °C for 10 min, followed by 40 cycles of 95 °C for 10 s, 60 °C for 45 s. These tissues were dissected from nine mussel individuals and pooled together to alleviate individual differentiation.

In the bacterial challenge experiment, live *V. alginolyticus* were used as the immune stimuli. A volume of 100 μL of bacteria dissolved in seawater (1 × 10^8^ CFU mL^−1^) was injected into the adductor of mussels. No injected mussels were used as the control. Nine mussels were randomly sampled from each group at 0, 3, 6, 12, 24, and 36 h post-challenge (hpc). The hemocytes from three mussels were pooled together to be deemed as one sample, and there were three samples for each time point. MicroRNAs were extracted using the miRNA Extraction Kit (HaiGENE, Cat. No.: B1802), and the cDNA was synthesized using the miRcute Plus miRNA First-Strand cDNA Kit (Tiangen, Cat. No.: 4992786) and qPCR was performed using the miRcute Plus miRNA qPCR Kit (Tiangen, Cat. No.: 4992887). U6 and β-actin genes were used as the internal references for qPCR and miRNAs qPCR, respectively. The specific primer pairs used in this experiment are listed in Table 1. The relative expression levels were measured using the 2^−ΔΔCt^ method [67].

### 4.6. Cell Culture

The mammalian HEK293 cells (RiboBio Ltd., Guangzhou, China) were employed to perform the luciferase reporter assays to determine the downstream activation by *Mc*IL-17-3 as well as the association between *Mc*-novel_miR_145 and *Mc*IL-17-3. HEK293 cells were cultured in OPTI-MEM medium (GIBCO) at 37 °C, 5% CO_2_. To further explore the regulation of *Mc*IL-17-3 by *Mc*-novel_miR_145 and their functional role in LPS-induced apoptosis, hemocytes of *M. coruscus* were retrieved from the adductor muscle of each mussel with a 0.5 mm-diameter (25 G) disposable needle containing 0.5 mL of the anticoagulant. Hemocytes were collected by centrifugation for 5 min at 3000 rpm, 4 °C, and 0.25% trypsin (Solarbio) was added. Hemocytes were suspended in an L-15 medium containing 15% fetal bovine serum (Solarbio) and cultured at 26 °C with 5% CO_2_.

### 4.7. Synthesis of miRNA Mimic and Inhibitor

The *Mc*-novel_miR_145 mimic, inhibitor, and control nucleotides were composed by GenePharma (Shanghai). Their sequence is as follows: *Mc*-novel_miR_145 mimic, 52032-UCCAGAAAAGCGCUUCGGACG-3′; *Mc*-novel_miR_145 inhibitor, 5′-CGUCCGAAGCGCUUUUCUGGA-3′ (chemically modified by 2′Ome); negative control mimic, 5′-UUGUACUACACAAAAGUACUG-3′; and negative control inhibitor, 5′-CAGUACUUUUGUGUAGUACAA-3′ (chemically modified by 2′Ome).

### 4.8. Luciferase Reporter Analysis

Luciferase reporter assays are widely used to study gene expression and regulatory activity. The amount of light generated is proportional to the amount of luciferase enzyme produced, which in turn reflects the activity of the regulatory element. The luciferase activity is then measured using a luminometer, and the results are analyzed and interpreted to determine the regulatory activity of the studied element. Luciferase reporter assays were employed to analyze the downstream activation of *Mc*IL-17-3. In this experiment, the pEGFP-*Mc*IL-17-3 plasmid (0.1, 0.5, and 1.0 µg/well) along with the pGLNF-κb-luc reporter plasmid (0.25 µg/well) were cotransfected into HEK293 cells using Lipo6000^TM^ for 24 h. A blank pEGFP-N1 plasmid was used as the control. Luciferase activity was measured using the Dual-Luciferase^®^ Reporter Assay System (Promega, Madison, WI, USA) according to the specification, with Renilla luciferase used as the inter control. *Mc*-novel_miR_145 targeting *Mc*IL-17-3 was also confirmed by luciferase reporter assays. The bioinformatic calculation predicted that *Mc*-novel_miR_145 can target the 3′-UTR of *Mc*IL-17-3, and subsequently cloned the 3′-UTR of *Mc*IL-17-3 into the pmiR-RB-Report^TM^ luciferase reporter vector to construct the wild *Mc*IL-17-3-3′UTR-WT reporter plasmid. The mutant-type *Mc*IL-17-3-3′UTR-MUT reporter vector was constructed by mutating the nucleotide at 1023–1040 sites: TCCGAAGCGCTTTTCTGG to AGGCTTCGCGAAAAGACC. RNA oligo (*Mc*-novel_miR_196 NC, mimic, NCi, inhibitor) was transfected along with *Mc*IL-17-3-3′UTR-WT or *Mc*IL-17-3-3′UTR-MUT into HEK293 cells using Lipo6000^TM^.

### 4.9. Recombinant Expression, Purification and the Antiserum Preparation

To assess the effect of *Mc*-novel_miR_145 on the expression of the *Mc*IL-17-3 protein, an antiserum of McIL-17-3 was prepared. The cDNA fragment covering the open reading frame (ORF) of *Mc*IL-17-3 was amplified with one specific primer pair (Table 1) and inserted into the pET-32a vector. The recombinant plasmid pET-32a-*Mc*IL-17-3 was transformed into Escherichia coli (DE3) (Takara) and incubated in LB medium (containing 50 mg L^−1^ kanamycin) at 37 °C with shaking at 130 rpm for 4 h. After the optical density reached absorbance 0.6 at 600 nm, the isopropyl-beta-D-thiogalactopy ranoside (IPTG) with a final concentration of 1 mM was added to the bacterial solution to induce the expression of recombinant *Mc*IL-17-3 protein (r*Mc*IL-17-3). After incubation at 37 °C for 6 h, the medium was centrifuged at 8000 rpm for 30 min to collect the bacteria, followed by suspension in TBS buffer (20 mM Tris-HCl, 150 mM NaCl, pH 8.0). The r*Mc*IL-17-3 was purified by Ni-nitorilotriacetic acid (NI-NTA) affinity chromatography, and the purified protein was dialyzed out of imidazole for 24 h. The resultant protein was isolated by reducing 12% SDS-polyacrylamide gel electrophoresis (SDS-PAGE). The purified protein was refolded in a gradient urea-TBS glycerol buffer (50 mM Tris-HCl, 50 mM NaCl, 2 mM reduced glutathione, 10% glycerol, 0.2 mM oxide glutathione, a gradient urea concentration of 6, 4, 3, 2, 1, and 0 M urea in each gradient, pH 7.4, each gradient at 4 °C for 12 h). Then, the resultant protein was used to immunize 6-week-old mice to acquire polyclonal antibodies.

### 4.10. Western Blotting

Protein samples were extracted from hemocytes with RIPA lysis buffer (Beyotime), and then the concentration was detected with a BCA kit. After isolation by SDS-PAGE, the protein was transferred to PVDF membranes with sealing by 5% skim milk powder in TBST (20 Mm Tris-HCl, 150 mM NaCl, 1% Tween-20, pH 8.0), followed by the antibody against *Mc*IL-17-3 incubation overnight. Subsequently, the membranes were incubated with a diluted solution of goat anti-mouse IgG antibody and alkaline phosphatase conjugate (Thermo Fisher Scientific, Cat. No.: 31324) in the secondary antibody dilution buffer (Beyotime, P0258) for 3 h. Finally, the immunoreactive proteins were detected by the ECL detection system.

### 4.11. Apoptosis

Hemocyte apoptosis was assessed using flow cytometry according to the manual of the FITC-Annexin-V Apoptosis Detection Kit (Beyotime). Briefly, the collected hemocytes were treated for 24 h with LPS (0.1 mg/mL), 20 nM of pEGFP-*Mc*IL-17-3 plasmid, *Mc*-novel_miR_145 mimic, and *Mc*-novel_miR_145 inhibitor. After washing with PBS, the cells were re-suspended in the L15 medium at a final concentration of 1 × 10^6^ cells mL^−1^ and were stained with FITC-Annexin-V and PI by being incubated at room temperature for 25 min in the dark. Finally, the flow cytometry instrument (Beckman CytoFLEX FCM) was employed to detect cell apoptosis, and data were analyzed using FlowJo^TM^ 10 software.

### 4.12. Statistical Analysis

Experimental results were presented as the mean ± standard deviation (S.D.). The results were processed using a two-way ANOVA analysis of variance with Tukey’s multiple comparisons test, and the Origin2021 software was employed to analyze the data and construct figures.

## 5. Conclusions

In this study, a novel IL-17 homolog, McIL-17-3, was identified from *M. coruscus* and found to play a crucial role in molluscan immune defense against bacterial attack. Moreover, it was discovered that *Mc*IL-17-3 is negatively regulated by *Mc*-novel_miR_145, which contributes to its participation in LPS-induced apoptosis. The results of this study provide valuable insights into the regulatory role of IL-17 in the immune response of mussels and highlight the potential of noncoding RNA in regulating invertebrate immune defense mechanisms. However, it is important to note that some limitations exist in this study. Specifically, the mechanism underlying the action mode of *Mc*IL-17-3 was not studied and requires further investigation. Additionally, this study only focused on the interaction between *Mc*-novel_miR_145 and *Mc*IL-17-3 and did not explore the possible involvement of other miRNAs in the immune response. Overall, this study emphasizes the important role of noncoding RNA in immune defense mechanisms and suggests that their modulation could serve as a potential strategy for targeted therapies for various diseases.

## Figures and Tables

**Table 1:** PCR primer pairs used in the present study.

Primer	Sequences (5′–3′)	Usage
U6	CTCGCTTCGGCAGCACA	Internal reference for miRNAs qPCR
	AACGCTTCACGAATTTGCGT	
β-actin	ATGAAACCACCTACAACAGT	Internal reference for qPCR
	TAGACCCACCAATCCAGACG	
*Mc*IL-17-3	TGCTCATTTGGTAGATCACGGA	For *Mc*IL-17-3 qPCR
	GCACTGTATGGCGTTTGCTC	
*Mc*-Novel_miR_145-F	ACACTCCAGCTGGGUCCAGAAAAGCGCUU	For *Mc*-novel_miR_145 qPCR
*Mc*IL-17-3-F	AAGGATCCATGTATTTTATCAATATACTTA	For pEGFP-*Mc*IL-17-3 plasmid construction
*Mc*IL-17-3-R	CCGCTCGAGTTCTTCTGGTCCATCAGCTGGA	
*Mc*IL-17-3-YF	CACGAATTCATGTATTTTATCAATATACTTA	For pET32a-*Mc*IL-17-3 plasmid construction
*Mc*IL-17-3-YR	GACGGATCCTTCTTCTGGTCCATCAGCTGGA	
*Mc*IL-17-3-3′UTR-WT-F	CCGCTCGAGTATGTGAACAGCCAAGAGAAGTCGCAAATGATGTA	For *Mc*IL-17-3-3′UTR-WT plasmid construction
*Mc*IL-17-3-3′UTR-WT-R	GCGGCCGCAAACAGAATACAAAAAACCCTTTATATGGCAAGTTG	

## Data Availability

Requests for access to the data, statistical code, questionnaires, and technical processes may be made by contacting the corresponding author at qpz2004@vip.sina.com, qipengzhi@zjou.edu.cn.
